# *Corallina* and *Ellisolandia* (Corallinales, Rhodophyta) photophysiology over daylight tidal emersion: interactions with irradiance, temperature and carbonate chemistry

**DOI:** 10.1007/s00227-014-2485-8

**Published:** 2014-07-27

**Authors:** C. J. Williamson, J. Brodie, B. Goss, M. Yallop, S. Lee, R. Perkins

**Affiliations:** 1 School of Earth and Ocean Sciences, Cardiff University, Park Place, Cardiff, CF10 3YE UK; 2 Department of Life Sciences, The Natural History Museum, Cromwell Road, London, SW7 5BD UK; 3 School of Biological Sciences, University of Bristol, Bristol, BS8 1UG UK

## Abstract

The photophysiology of three geniculate coralline algal species (*Corallina officinalis*, *C. caespitosa* and *Ellisolandia elongata*) was determined in intertidal rock pools in the south-west UK at Combe Martin (51°12′31N 4°2′19W) and Heybrook Bay (50°31′66N 4°11′41W), at the start, middle and end of summer (September 1 and 2) and winter (February 9 and 10) daylight tidal emersion periods, in relation to prevailing irradiance, temperature and carbonate chemistry conditions. Algal photophysiology was assessed from rapid light curves performed using pulse amplitude modulation fluorometry. *Corallina* and *Ellisolandia* experienced significant fluctuations in irradiance, temperature and carbonate chemistry over seasonal and tidal cycles. Rock pool carbonate chemistry was predictable (*R* ^2^ = 0.82, *P* < 0.0001) by photodose (summed irradiance) plus water temperature, but not significantly related to photophysiology. In contrast, *Corallina* and *Ellisolandia* relative maximum electron transfer rate showed a significant negative relationship (*R* ^2^ = 0.65, *P* < 0.0001) with irradiance plus water temperature. At a seasonal resolution, photoacclimation to maximize both light harvesting during winter months and photoprotection during summer months was observed for all species. Dynamic photoinhibition was apparent over both summer and winter tidal emersion, in relation to irradiance fluctuations. More effective photoinhibition was apparent during summer months, with greater sensitivity to irradiance and slower recovery in *F*_v_/*F*_m_, observed during winter. With sustained high irradiance over tidal emersion, the establishment of high pH/low inorganic carbon conditions may impact photochemistry. This study represents the first assessment of *C. officinalis*, *C. caespitosa* and *E. elongata* photophysiology underpinned by clear species concepts and highlights their ability to adapt to the dramatically fluctuating conditions experienced in intertidal rock pools.

## Introduction

Calcified macroalgae are particularly ecologically important in shallow temperate regions (Johansen 1981). Acting as ecosystem engineers (sensu Jones et al. 1994), they provide habitat for numerous small invertebrates, shelter from the stresses of intertidal life via their physical structure, and surfaces for the settlement of microphytobenthos (see Nelson 2009 for a full review). The Corallinales are the predominant order of calcified macroalgae found in temperate waters and comprise both non-genicluate genera that are mostly encrusting and turf forming geniculate genera (Irvine and Chamberlain 1994; Nelson 2009). In the UK intertidal, turfing species of the genera *Corallina* and *Ellisolandia* are epilithic on both exposed substrata and in rock pool habitats (Brodie et al. 2013), where they must tolerate significant fluctuations in abiotic conditions including irradiance, temperature and rock pool water chemistry (Ganning 1971; Truchot and Duhamel-Jouve 1980; Morris and Taylor 1983).

Irradiance is one of the most important factors controlling the distribution of macroalgae in the littoral zone and also one of the most complex (Luning 1990; Lobban and Harrison 1994). Large fluctuations occur diurnally because of changes in cloud cover, tides and the angle of the sun, and both predictable variability (changes in day length and solar angle) and unpredictable (cloudiness, turbidity and run-off) variability are observed seasonally (Lobban and Harrison 1994). Within the intertidal, sessile macroalgae have to cope with the changing irradiance regime, facing serious photostress during tidal emersion when exposed to high irradiances (Davison and Pearson 1996; Häder et al. 1997; Franklin and Forster 1997). Production of reactive oxygen species as by-products of photosynthesis is increased under high irradiance, causing photooxidative damage, which can ultimately lead to pigment bleaching and death (Muller et al. 2001). Macroalgae have thus developed regulatory mechanisms to ameliorate light stress, including adjustment of the antenna size, thermal dissipation of excess excitation energy, antioxidant systems and the fast repair of photooxidative damage (Häder et al. 2003).

Temperature is also a key factor governing both the large-scale geographical distribution of macroalgal species and the small-scale vertical distribution of species on a shore (Luning 1990) and is of high importance when discussing rock pool ecology (Ganning 1971). In rock pools, temperature is closely related to local climate, especially air and ambient seawater temperature, irradiance, wind, the time of day at which low tide occurs and the extent of heating or cooling due to wave action (Ganning 1971; Lobban and Harrison 1994). At the level of the individual, temperature has fundamental effects on chemical reaction rates and, in turn, metabolic pathways, with complex interactions with other factors (Lobban and Harrison 1994). For example, in photosynthesis, diffusion rates, carbonic anhydrase (CA) activity and active transport of CO_2_ and HCO_3_^−^ are all affected by temperature, and thus temperature will influence the supply of substrate to carbon fixation pathways (Lobban and Harrison 1994).

It has long been established that fluctuations in rock pool water chemistry are apparent due to the interactions between physio-chemical and biological processes (Ganning 1971; Daniel and Boyden 1975; Morris and Taylor 1983). Truchot and Duhamel-Jouve (1980) provided the first analysis of diurnal changes taking place in the carbonate system of rock pools, and Morris and Taylor (1983) extended this work to examine both diurnal and seasonal changes, demonstrating that diurnal fluctuations in *p*O_2_, *p*CO_2_ and pH were directly related to the photosynthetic activity of the pool flora and to the respiration of both flora and fauna (Morris and Taylor 1983). More recently, interactions between the carbonate system of seawater and the photosynthesis of macroalgae have been examined. The absence of certain macroalgal species from rock pool habitats has, for example, been attributed to the establishment of adverse high pH and low inorganic carbon (Ci) conditions due to the photosynthetic utilization of Ci by *Ulva intestinalis* in Swedish rock pools (Björk et al. 2004). In shallow water macroalgal habitats (0–1 m), high pH has also been shown to have a direct negative effect on the photosynthesis of *Fucus vesiculosus*, *F. serratus*, *Ceramium rubrum* and *Ulva* sp., not accounted for alone by the low availability of Ci (Middelboe and Hansen 2007a).

Variability in carbonate chemistry is also important with regard to species’ responses to future ocean acidification (OA) (Hofmann et al. 2011; Andersson and Mackenzie 2012; Hofmann et al. 2014). With OA, increasing concentrations of dissolved CO_2_ are shifting the seawater carbonate chemistry equilibrium, increasing hydrogen ion (H^+^) and bicarbonate (HCO_3_^−^) concentrations, and subsequently decreasing the concentration of carbonate (CO_3_^2−^) available for calcification (Doney 2006; Cao et al. 2007; Doney 2010). These changes are predicted to pose significant negative impacts to calcifying macroalgal species (Harley et al. 2012). As OA proceeds, however, periodic exposure to high pH conditions may ameliorate some of the negative impacts on calcifying species (Hurd et al. 2011; Anthony et al. 2011; Manzello et al. 2012). In addition, local adaptation of calcifying species to natural pH variability has been linked to increased resilience to future OA conditions (Wootton et al. 2008; Hofmann et al. 2011; Kelly et al. 2013; Wolfe et al. 2013; Hofmann et al. 2014).

The aim of the present study was to provide an assessment of the in situ photophysiology of three turfing geniculate coralline algal species, *Corallina officinalis, C. caespitosa* and *Ellisolandia elongata*, within rock pool habitats, in relation to the irradiance, temperature and carbonate chemistry conditions prevailing over tidal emersion periods. Recent molecular insights into cryptic diversity within the genus *Corallina* has resulted in (1) the splitting of the well-known *C. officinalis* into two genetically distinct species, *C. officinalis* and *C. caespitosa* (Walker et al. 2009), (2) a revised definition of *C. officinalis* and *C. elongata* (Brodie et al. 2013) and (3) the establishment of a new genus, *Ellisolandia,* containing a single species, *E. elongata*, previously *Corallina elongata* (Hind and Saunders 2013). As such, almost no information is currently available on the ecology of *C. caespitosa* [though, see Williamson et al. (in review) and Brodie et al. (2013)], which was likely previously investigated under the name *C. officinalis*, particularly if originating from outside of the NE Atlantic (Williamson et al. in review). These phylogenetic advances allow for an examination of the three species’ ecology, underpinned by clear species concepts. In addition, while recent research has examined the potential impacts of OA on *C. officinalis* and *E. elongata* (Egilsdottir et al. 2013; Hofmann et al. 2012, 2013; Noisette et al. 2013), we still lack a decent understanding of the present-day ecology of these species in situ, particularly in relation to abiotic parameters that will significantly change under a high CO_2_ world, i.e. temperature and carbonate chemistry.

Observations for the present study were conducted over summer and winter daylight tidal emersion periods at two south-westerly UK intertidal sites. Rapid light curves (RLCs) were performed using pulse amplitude modulation (PAM) fluorometry to assess the actual photophysiology of algae at the time of sampling (Ralph and Gademann 2005; Perkins et al. 2010), as opposed to the theoretical potential of photochemistry, facilitating comparison to ambient irradiance and rock pool water temperature and carbonate chemistry monitored in parallel.

## Methods

### Study sites and species distributions

**Fig. 1:**
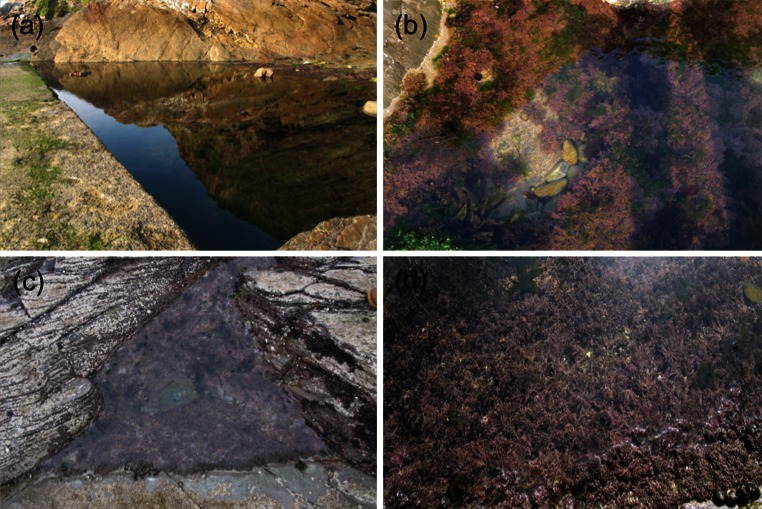
Examples of sampled rock pools and associated *Corallina* assemblages. **a** A typical large upper shore rock pool at Combe Martin (Pool 1) created by man-made walkway, **b** showing rock pool assemblage during summer; **c** smaller lower shore rock pool at Heybrook Bay (Pool 1), **d** showing rock pool assemblage during winter. See Table 1 for rock pool attributes

The photophysiology of *Corallina officinalis*, *C. caespitosa* and *Ellisolandia elongata* and the irradiance, water temperature and carbonate chemistry conditions were monitored over daylight tidal emersion periods during summer (1/2 September 2012) and winter (9/10 February 2013), at upper shore Combe Martin (CM), North Devon and upper and lower shore Heybrook Bay (HB), South Devon, UK (Fig. 1; Table 1). All sampling was performed on or ±1 day of spring tides to allow observation of potential extremes in summer and winter photophysiology and abiotic parameters.

**Table 1:** Site and sampling details

	Combe Martin	Heybrook Bay	
Location	51°12′31N 4°2′19W	50°31′66N 4°11′41W	
Tidal range	MHWS–MLWS = 9.2 − 0.68 (8.52) MHWN–MLWN = 6.9 − 3.1 (3.8)	MHWS–MLWS = 5.5 − 0.8 (4.7) MHWN–MLWN = 4.4 − 2.2 (2.2)	
Summer sampling date	02.09.12	01.09.12	
Summer tides	06:48 = 9.2/12:49 = 1.1/19:05 = 9.4	06:03 = 5.4/12:12 = 0.6/18:16 = 5.6	
Winter sampling date	10.02.13	09.02.13	
Winter tides	05:51 = 9.5/12:00 = 0.8/18:15 = 9.4	04:46 = 5.4/11:12 = 0.8/17:18 = 5.3	
Shore height sampled	Upper	Upper	Lower
*Corallina* spp. present	*C. officinalis, C. caespitosa*	*C. officinalis*	*C. officinalis, C. elongata*

Pool	1	2	3	1	2	3	1	2	3
Height above chart datum (m)	5.61	5.77	5.32	4.24	4.08	3.30	2.08	2.34	2.30
Volume (m^3^)	82.85	201.4	61.14	0.58	0.35	0.40	0.10	1.31	2.06
Surface area (m^2^)	165.7	493.74	181.45	5.14	3.43	2.92	0.80	13.68	15.39
Maximum depth (cm)	67.0	80.0	71.0	28.9	13.8	21.5	40.6	19.8	19.8

Tidal range demonstrates mean high/low water spring/neap expressed as range (m). Summer and winter tides report the time and tidal height (m) of high/low/high tides prevailing on sampling days. Lower table summarizes rock pool attributes

*Corallina officinalis* is widely distributed around the entire UK, while *C. caespitosa* appears to be more southerly, only occurring on shores in England, and *Ellisolandia elongata* demonstrates a westerly distribution. At study sites, *C. officinalis* is present at both CM and HB occurring from the lower to the upper shore. *Corallina caespitosa* is present in upper shore rock pools at CM, where it inhabits a narrow zone (ca. 2 cm) at the upper water line of rock pools, *C. officinalis* dominating below this zone. Of the study sites, *E. elongata* is present in suitable abundances for the present study at lower shore HB only. Field studies were therefore performed at upper shore CM (*C. officinalis* and *C. caespitosa* present) and upper (*C. officinalis* present) and lower shore (*C. officinalis* and *E. elongata* present) HB, to allow assessment of the three desired species. Species identification was verified by extraction and amplification of the COI gene region and comparison to published sequences of the three species as per Walker et al. (2009) and Brodie et al. (2013).

At upper shore in both CM and HB, *Corallina* photophysiology and abiotic conditions were monitored at the start, middle and end of tidal emersion periods in three rock pools (Fig. 1; Table 1). Start and end of the emersion period were defined as being within 30 min of tidal isolation (start of emersion) and tidal reconnection (end of emersion) of the rock pool to the main tidal water mass. Mid emersion period was defined as the time midway between the start and end of emersion measurements. At lower shore HB, *Corallina* and *Ellisolandia* photophysiology and abiotic conditions were monitored in three rock pools at the start and end of tidal emersion periods only, given the shorter duration of tidal emersion at this shore height. In all cases, rock pools were selected where *Corallina* and/or *Ellisolandia* demonstrated >ca. 75 % cover, visually estimated by the authors.

### Monitoring of abiotic conditions

Ambient photosynthetically active radiation (PAR, μmol photons m^−2^ s^−1^) was measured three times during each sampling period [start, middle (upper shore only) and end of emersion] per rock pool (total *n* = 9 measurements per emersion period), using a 2 pi LI-COR cosine-corrected quantum sensor positioned ca. 5 cm above the surface of the rock pools. For each recording, a 15-s average was taken using an automated function on the sensor. The average irradiance for the start, middle and end periods of tidal emersion was calculated as the average of all measurements taken across respective sampling periods for all pools. Cumulative photodose (PAR, mol photons m^−2^) was calculated from irradiance measurements by summing PAR over time from the start of tidal emersion of rock pools and calculation to more appropriate units. In parallel, rock pool water temperatures were monitored with a digital thermometer as above.

Collection of water samples for determination of carbonate chemistry followed the methods of Dickson et al. (2007) adapted for coastal fieldwork. During each period of tidal emersion [start, middle (upper shore only), and end], two water samples were collected in 250-ml borosilicate glass bottles (Schott Duran) from approximately 5-cm depth in the centre of each rock pool. 1 % volume (2.5 ml) was discarded to allow for water expansion, and 0.02 % by volume (50 μl) of saturated mercuric chloride solution was added to poison the sample. Bottles were immediately closed and sealed with pre-greased, ground-glass stoppers to ensure gas-tight conditions, and bound with electrical tape. Samples were stored in a cool (approximately 4–6 °C), dark (no ambient detectable light) location until analysis.

Carbonate chemistry parameters, *p*CO_2_, pH, HCO_3_^−^, CO_3_^2−^ and the saturation states of aragonite, *Ω*_arg_, and calcite, *Ω*_cal_, were determined from measurements of dissolved inorganic carbon (DIC) and total alkalinity (TA) performed on all carbonate chemistry water samples by the UK Ocean Acidification Carbonate Chemistry Facility at the National Oceanography Centre, Southampton, UK. DIC was analysed with an Apollo SciTech DIC analyzer (AS-C3), using a LI-COR (7000) CO_2_ infrared analyser. TA was determined using an open-cell titration (Dickson et al. 2007) with the Apollo SciTech’s AS-ALK2 Alkalinity Titrator. For both DIC and TA, the precision was 0.1 % or better and the accuracy was controlled against Certified Reference Materials (A.G. Dickson, Scripps). Carbonate chemistry parameters were calculated with CO2SYS (version 1.05, Pierrot et al. 2006), using the constants of Mehrbach et al. (1973) refitted by Dickson and Millero (1987).

### *Corallina* and *Ellisolandia* photophysiology

The photophysiology of *C. officinalis, C. caespitosa and E. elongata* was determined using PAM fluorometry. RLCs (Perkins et al. 2006) were performed using a Walz Water-PAM fluorometer, with three replicate light curves performed per *Corallina* and/or *Ellisolandia* species present in each rock pool (Table 1), at the start, middle (upper shore only) and end of summer and winter tidal emersion. Algal fronds were randomly selected from the upper 5 cm of rock pool walls for RLC analysis to allow some degree of continuity in light field experienced, with the exception of *C. caespitosa* at CM that is only found in a ca. 2 cm narrow zone along the upper water line of rock pool walls. RLCs were performed on the tips of fronds to avoid potentially self-shaded frond regions, and care was taken to determine RLCs on the side of fronds facing direct sunlight, as, e.g., the underside of fronds likely demonstrate differential photoacclimation.

RLCs are an effective tool with which to detect the operational photophysiology of a sample at the time measurements are made, providing information on the dissipation of energy from limiting levels of irradiance through to saturating levels, and can act as a proxy for the electron transport rate through photosystem II (Burdett et al. 2012). RLCs differ from traditional *P*–*I* curves in that they measure the actual, rather than the optimal, photosynthetic state, as steady state is not achieved during each light step duration (Ralph and Gademann 2005; Perkins et al. 2010).

RLCs were performed using a saturating pulse at a setting of ca. 8,600 μmol photons m^−2^ s^−1^ PAR, for 600 ms duration, and with nine 30 s incrementally increasing light steps from 0 to 1,944 μmol photons m^−2^ s^−1^ PAR. Light step duration was selected to balance potential photoacclimation occurring during longer light steps (60 s), with errors associated with shorter light steps (10 s) when samples have been exposed to high light (Perkins et al. 2006). Analysis of RLCs followed Perkins et al. (2006) with iterative curve fitting (Sigmaplot v. 14) and calculation of the relative maximum electron transfer rate (rETR_max_), the theoretical maximum light utilization coefficient (*α*) and the light saturation coefficient (*E*_*k*_) following Eilers and Peeters (1988). In addition, the approximate maximum light use efficiency in the dark-adapted state, the Genty parameter (Genty et al. 1989), was calculated as:

$$F_{\text{v}} /F_{\text{m}} = \left( {F_{\text{m}} - F_{\text{o}} } \right)/F_{\text{m}}$$

where *F*_m_ is the maximum yield, and *F*_o_ is the minimum fluorescence yield in the dark-adapted state. As long periods of dark adaption should be avoided prior to RLCs due to potential modification of the photoacclimation state of the cells investigated (Ralph and Gademann 2005; Perkins et al. 2010) and can be impractical when working under time constraints in situ (Burdett et al. 2012), *F*_v_/*F*_m_ was calculated from *F*_m_ and *F*_o_ values obtained during the initial light curve step of 30 s darkness. Burdett et al. (2012) demonstrated that a 10-s period was sufficient for the dark adaption of the red coralline alga *Lithothamnion* *glaciale* for in situ work, with F_v_/*F*_m_ 95–98 % of the maximum *F*_v_/*F*_m_ achieved after 5 min of darkness (=fully dark-adapted state). Our methodology thus allowed time constraints to be balanced when working over tidal emersion periods in situ, while allowing for sufficient dark adaptation of samples for RLC techniques (Ralph and Gademann 2005; Burdett et al. 2012).

### Data analysis

All statistical analyses and plotting of data were performed using R v.3.0.2 (R Core Team 2013). Prior to all analyses, normality of data was tested using the Shapiro–Wilk test and examination of frequency histograms. If data were not normally distributed, Box–Cox power transformation was applied using the boxcox function of the MASS package (Venables and Ripley 2002), and normality re-checked. Following the application of models to data as described below, model assumptions were checked by examination of model criticism plots.

### Abiotic environment

Differences in irradiance between seasons (summer and winter) and tidal emersion periods (start, middle and end) were examined for upper shore data, per site, using analysis of variance (ANOVA) with the fixed factors ‘Season’ (two levels), ‘Tide’ (three levels) and the interaction term ‘Season/Tide’. Post hoc Tukey honest significant differences analysis was performed on significant ANOVA results. Lower shore HB data were analysed as above though with two levels for the factor ‘Tide’. Differences in rock pool water temperatures were examined separately per site, using linear mixed-effects models with restricted maximum likelihood (REML) criterion, using the lmer function of package lme4 (Bates et al. 2013). Upper shore data were analysed with the fixed effects ‘Season’ (two levels), ‘Tide’ (three levels), the interaction term ‘Season/Tide’ and ‘Pool’ as random term (three levels). Lower shore HB data were examined in the same manner though with two levels for the fixed effect ‘Tide’. Upper- and lower-bound *P* values for the ANOVA were calculated for lmer models using the pamer.fnc function of the LMERConvenienceFunctions package (Tremblay and Ransijn 2013). Lower-bound *P* values (more conservative) and associated denominator degrees of freedom are reported. Post hoc analyses of significant differences highlighted by lmer models were performed using mcposthoc.fnc and summary.mcposthoc functions of the same package (Tremblay and Ransijn 2013).

All carbonate chemistry parameters were summarized using principal components analysis (PCA) with scaled variables. Differences in carbonate chemistry between seasons and over tidal emersion periods were examined by analysis of principal component one (PC1) and principal component two (PC2) using linear mixed-effects models as described above. Least squares multiple linear regression was used to examine relationships between PC1 and irradiance (analysed separately as both irradiance measured and calculated cumulative photodose) and rock pool water temperature. The relative importance of predictor variables was calculated using the calc.relimp function of relaimpo package using type ‘lmg’, whereby *R* ^2^ is partitioned by averaging over orders (Grömping 2006). Only statistically significant regressions are reported.

### Photophysiology

Differences in rETR_max_, *α*, *E*_*k*_ and *F*_v_/*F*_m_ were analysed separately per site, using linear mixed-effects models. CM upper shore data were analysed with the fixed effects ‘Season’ (two levels), ‘Tide’ (three levels), ‘Species’ (two levels), interaction terms ‘Season/Tide’ and ‘Species/Tide’, and the random term ‘Pool’ (three levels). HB upper shore data were analysed in the same manner though without the fixed effect ‘Species’ as only *C. officinalis* is present. HB lower shore data were analysed in the same manner with the exceptions of two levels for the fixed effect ‘Tide’. Calculation of *P* values and post hoc analyses were conducted as detailed above using the LMERConvenienceFunctions package (Tremblay and Ransijn 2013).

To examine relationships between *Corallina* spp. and *Ellisolandia* photophysiology and the prevailing abiotic conditions, rETR_max_, *α*, *E*_*k*_ and F_v_/*F*_m_ were regressed against irradiance (separately as both irradiance measured and calculated photodose), rock pool water temperature, PC1 and PC2, using least squares multiple linear regression as detailed above. Only statistically significant regressions are reported.

## Results

### Abiotic conditions

**Fig. 2:**
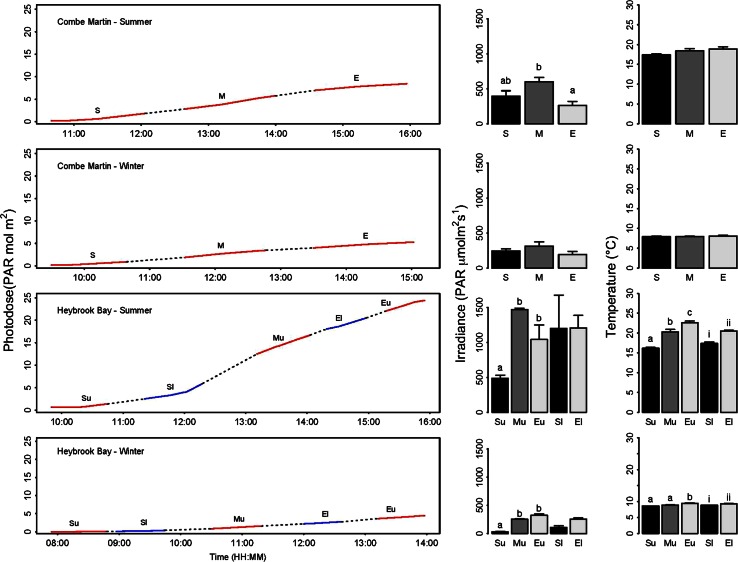
Photodose, irradiance and rock pool water temperature over summer and winter tidal emersion periods at CM and HB. Large plots display photodose as a function of time, with start (*S*), middle (*M*) and end (*E*) sampling times highlighted in *red* for CM and start (*Su*), middle (*Mu*) and end (*Eu*) upper shore sampling times highlighted in *red* for HB. Start (*Sl*) and end (*El*) lower shore sampling times at HB are indicted in *blue*. Middle and right columns represent average (*n* = 9 ± SE) irradiance and water temperature at the start, mid and end tidal emersion, respectively. *Letters* and *numerals* denote significant differences for upper and lower shore, respectively

Significantly higher irradiance was recorded during summer than winter at both CM (*F*_1,17_ = 10.07, *P* < 0.01) and upper/lower shore HB (*F*_1,17*/*1,11_ = 202.37/48.74, *P* < 0.001 in both cases) (Fig. 2). Significant differences in irradiance were also apparent over summer tidal emersion at CM (*F*_2,17_ = 6.78, *P* < 0.05), and both summer and winter tidal emersion at HB (*F*_2,17_ = 54.48, *P* < 0.0001), with significant interaction between ‘Season’ and ‘Tide’ (*F*_2,17_ = 6.025, *P* < 0.05). No significant difference in irradiance was evident between start and end tidal emersion at lower shore HB.

Rock pool water temperatures were significantly higher during summer than winter at CM (*F*_1,17_ = 2,408.30, *P* < 0.0001) and upper/lower shore HB (*F*_1,17*/*1,11_ = 2,900.70/3,927.52, *P* < 0.0001 in both cases) (Fig. 2). Over tidal emersion periods, no significant difference in water temperature was evident during either summer or winter at CM, while temperatures showed significant increases in both upper (*F*_2,17_ = 67.15, *P* < 0.0001) and lower shore (*F*_1,11_ = 85.75, *P* < 0.0001) rock pools at HB during both seasons (Fig. 2). The magnitude of increase in water temperature was greater during summer than winter at HB, as evidenced by significant interaction between ‘Season’ and ‘Tide’ for upper (*F*_2,17_ = 21.89, *P* < 0.0001) and lower shore (*F*_1,11_ = 14.77, *P* < 0.01) rock pools.

**Fig. 3:**
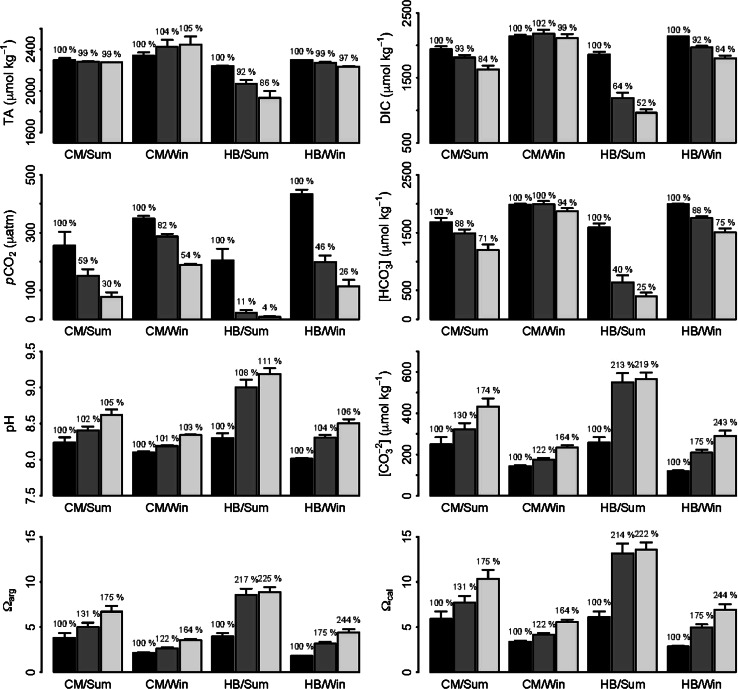
Average carbonate chemistry (TA, DIC, *p*CO_2_, HCO_3_^−^, pH, CO_3_^2−^, *Ω*_arg_ and *Ω*_cal_) recorded during summer (*Sum*) and winter (*Win*) at upper shore Combe Martin (*CM*) and Heybrook Bay (*HB*), at the start (*black bars*), middle (*dark grey bars*) and end (*light grey bars*) of tidal emersion periods (*n* = 6 ± SE). Percentages denote % change in parameters in relation to start values

**Fig. 4:**
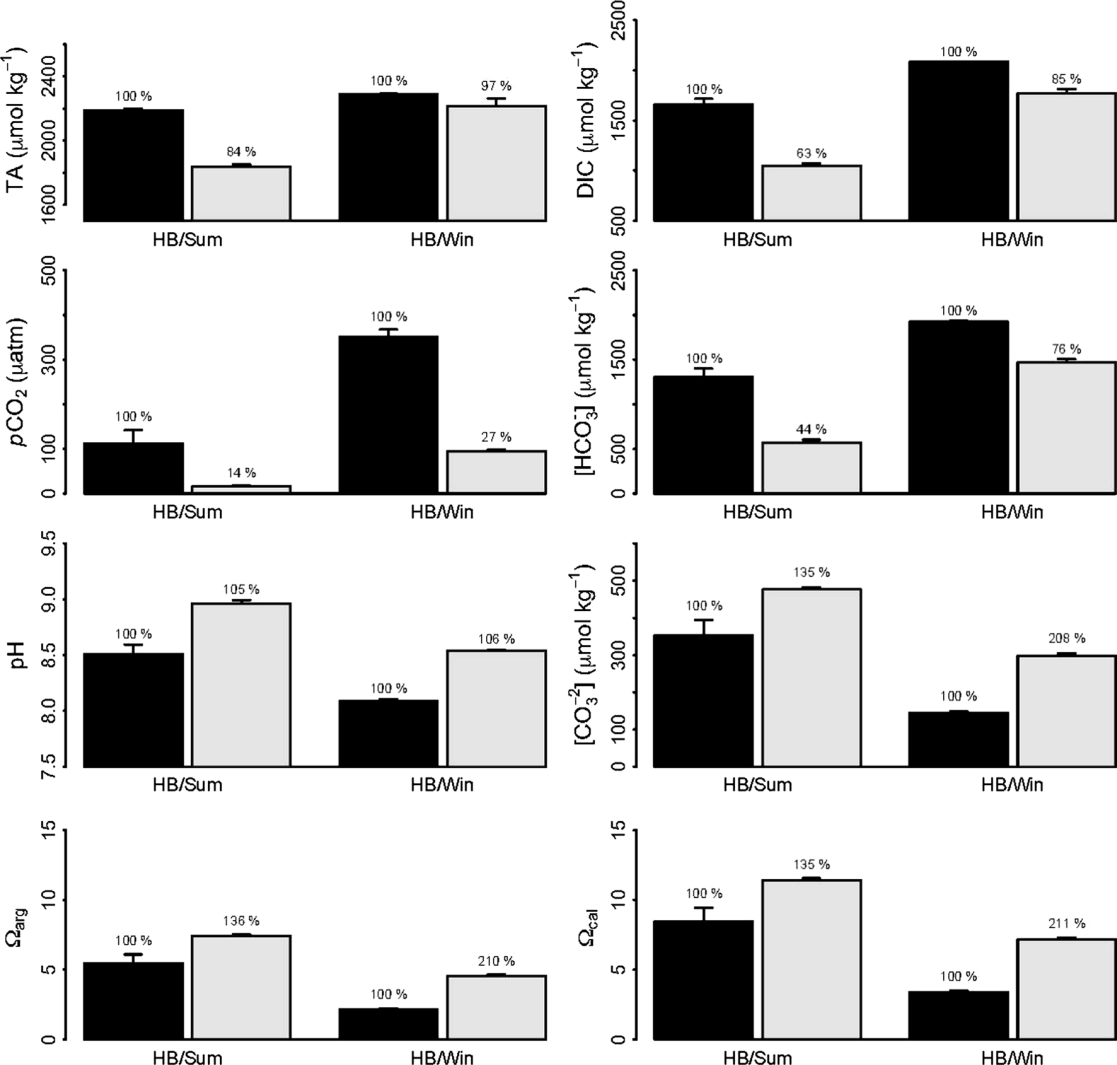
Average carbonate chemistry (TA, DIC, *p*CO_2_, HCO_3_^−^, pH, CO_3_^2−^, *Ω*_arg_ and *Ω*_cal_) recorded during summer (*Sum*) and winter (*Win*) at lower shore Hebrook Bay (*HB*), at the start (*black bars*) and end (*light grey bars*) of tidal emersion periods (*n* = 6 ± SE). Percentages denote % change in parameters in relation to start values

Changes in rock pool water carbonate chemistry were observed over both summer and winter daylight tidal emersion periods at CM and both upper and lower shore HB (Figs. 3, 4). *p*CO_2_ and HCO_3_^−^ decreased over tidal emersion, with concomitant increases in pH, CO_3_^2−^, *Ω*_arg_ and *Ω*_cal_. The greatest magnitude of change in carbonate chemistry was observed over summer tidal emersion in upper shore rock pools at HB, with *p*CO_2_ and HCO_3_^−^ concentrations decreasing to 4 and 25 % of start values, respectively, and pH increasing to 111 %, and CO_3_^2−^, *Ω*_arg_ and *Ω*_cal_ all increasing to ca. 220 % of start values, by the end of tidal emersion.

**Fig. 5:**
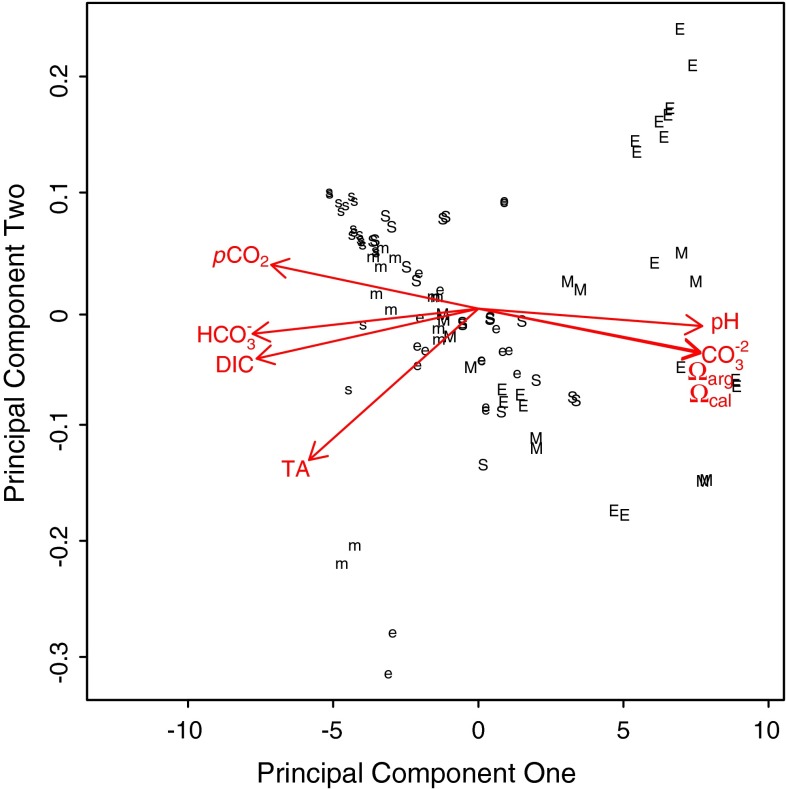
Principal components analysis of carbonate chemistry parameters, showing principal component one (89.3 % of variance) in relation to principal component two (8.2 % of variance). Summer data are indicated by *upper case letters* and winter by *lower case letters*, representing the start (*s*), mid (*m*) and end (*e*) of tidal emersion periods

**Fig. 6:**
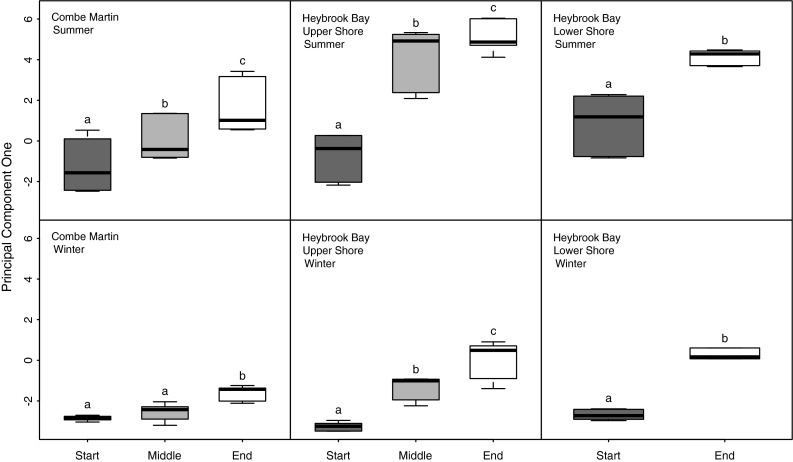
Boxplots showing the median, minimum, maximum and first and third quartiles of principal component one in relation to tidal emersion period at upper shore Combe Martin and upper and lower shore Heybrook bay. *Letters* denote significant difference

PCA served to summarize all carbonate chemistry parameters for subsequent analysis (Table 2; Fig. 5). PC1 described 89.3 % of the variance in carbonate chemistry data and represented changes in rock pool carbonate chemistry observed over tidal emersion periods, i.e. a shift from high *p*CO_2_, HCO_3_^−^ and high overall DIC, to high pH, CO_3_^2−^, *Ω*_arg_ and *Ω*_cal_ (Table 2; Fig. 5). PC2 accounted for 8.2 % of the variance and, mainly, represented differences in TA within the data (Table 2; Fig. 5). Significantly higher values of PC1 were observed for summer data than winter data for CM (*F*_1,32_ = 94.92, *P* < 0.0001), and upper (*F*_1,32_ = 767.30, *P* < 0.0001) and lower shore HB (*F*_1,20_ = 165.14, *P* < 0.0001) (Fig. 6). PC1 also showed significant increases over tidal emersion periods during both summer and winter for CM (*F*_2,32_ = 22.18, *P* < 0.0001), and upper (*F*_2,32_ = 345.72, *P* < 0.0001) and lower shore HB (*F*_1,20_ = 119.35, *P* < 0.0001) (Fig. 6). The magnitude of increase in PC1 was greater during summer than winter for HB upper shore, as shown by significant interaction between ‘Season’ and ‘Tide’ (*F*_2,32_ = 9.38, *P* < 0.0001).

**Table 2:** Component loadings of principal components analysis of carbonate chemistry parameters (TA, DIC, pH, *p*CO_2_, HCO_3_^−^, CO_3_^2−^, *Ω*_arg_ and *Ω*_cal_)

	PC1 (%)	PC2 (%)	PC3 (%)
Proportion of variance	89.3	8.2	2.0
Cumulative proportion	89.3	97.5	99.5

Variable	PC1	PC2	PC3
*Component loadings*			
TA	−0.27	−0.81	0.12
DIC	−0.36	−0.27	−0.11
pH	0.36	−0.09	0.01
*p*CO_2_	−0.34	0.23	0.89
HCO_3_^−^	−0.37	−0.13	−0.15
CO_3_^2−^	0.36	−0.24	0.20
*Ω*_arg_	0.36	−0.23	0.23
*Ω*_cal_	0.36	−0.24	0.21

**Fig. 7:**
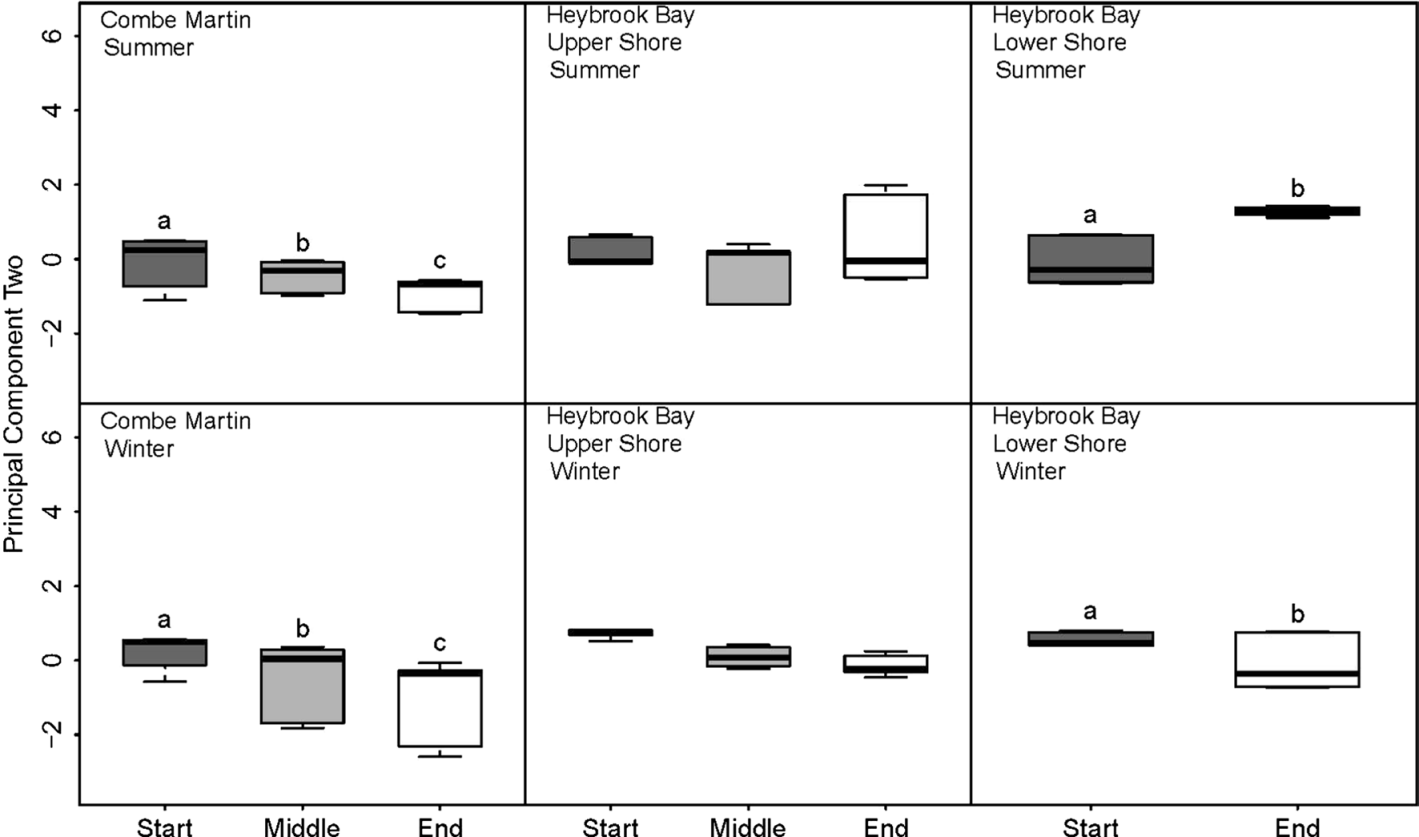
Boxplots showing the median, minimum, maximum and first and third quartiles of principal component two in relation to tidal emersion period at upper shore Combe Martin and upper and lower shore Heybrook bay. *Letters* denote significant difference

PC2, mainly representing TA within the dataset, was not significantly different between seasons for CM or upper shore HB, though it was significantly different between summer and winter for lower shore HB (*F*_1,20_ = 15.64, *P* < 0.01) (Fig. 7). Over tidal emersion periods, PC2 significantly decreased for CM upper shore during both summer and winter (*F*_2,32_ = 28.37, *P* < 0.0001). PC2 was also significantly different between start and end lower shore HB tidal emersion (*F*_1,20_ = 15.61, *P* < 0.01), with the direction of change different during summer and winter, as highlighted by significant interaction between ‘Season’ and ‘Tide’ (*F*_1,20_ = 92.03, *P* < 0.0001). While no significant difference in PC2 was observed for HB in relation to ‘Tide’, there was a significant interaction between ‘Season’ and ‘Tide’ (*F*_2,32_ = 3.85, *P* < 0.05).

**Fig. 8:**
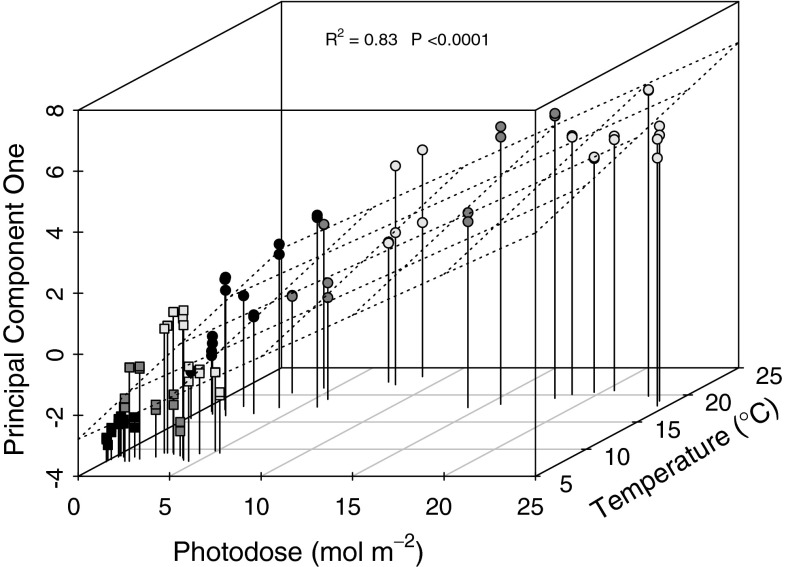
Multiple linear regression of principal component one in relation to photodose (67 % relative importance) and rock pool water temperature (32 % relative importance). *Dashed grid* demonstrates the regression plane. Summer (*circles*) and winter (*squares*) data are highlighted in relation to start (*black*), middle (*dark grey*) and end (*light grey*) tidal emersion

Least squares multiple linear regression identified significant relationships between carbonate chemistry (as PC1) in relation to photodose and rock pool water temperature (*R* ^2^ = 0.82, *P* < 0.0001) (Table 3; Fig. 8). The relative importance of predictors was given as 67 % for photodose and 32 % for temperature, respectively.

**Table 3:** Multiple linear regression analysis of principal component one (PC1) in relation to irradiance (as cumulative photodose) and rock pool water temperature (Temp.), showing associated standard error (SE) of coefficients, the significance of predictor variables within the model (Pred. sig.), the relative importance of predictor variables (Rel. imp.), associated overall model *R* ^2^ and significance (Model *P*), and the number of observations (*n*)

Relationship (*Y* = *a* + *b*_1_*X_1_ + *b*_2_*X_2_)	Coefficient SE			Pred. sig.		Rel. imp.		*R* ^2^	Model *P*	*n*
	*a*	*b*_1_	*b*_2_	*X*_1_	*X*_2_	*X*_1_	*X*_2_			
PC1 = − 3.456 + 0.270 Photodose + 0.134 Temp	0.321	0.019	0.025	<0.0001	<0.0001	67 %	32 %	0.83	<0.0001	96

### *Corallina* and *Ellisolandia* photophysiology

**Fig. 9:**
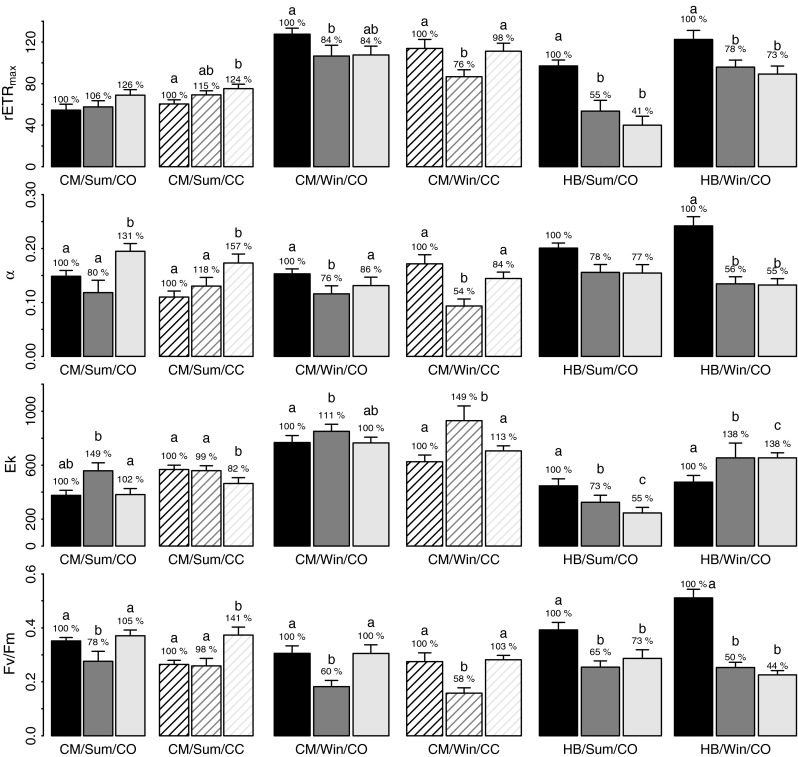
Average *Corallina officinalis* (*CO*—*filled bars*), and *Corallina caespitosa* (*CC*—*lined bars*) rETR_max_, *α*, *E*_*k*_ and *F*_v_/*F*_m_ at upper shore Combe Martin (*CM*) and Heybrook Bay (HB) at the start (*black bars*), middle (*dark grey bars*) and end (*light grey bars*) of summer (*Sum*) and winter (*Win*) tidal emersion periods (*n* = 9 ± SE). Percentages demonstrate % change in parameters normalized to start emersion values. *Letters* denote significant differences

**Fig. 10:**
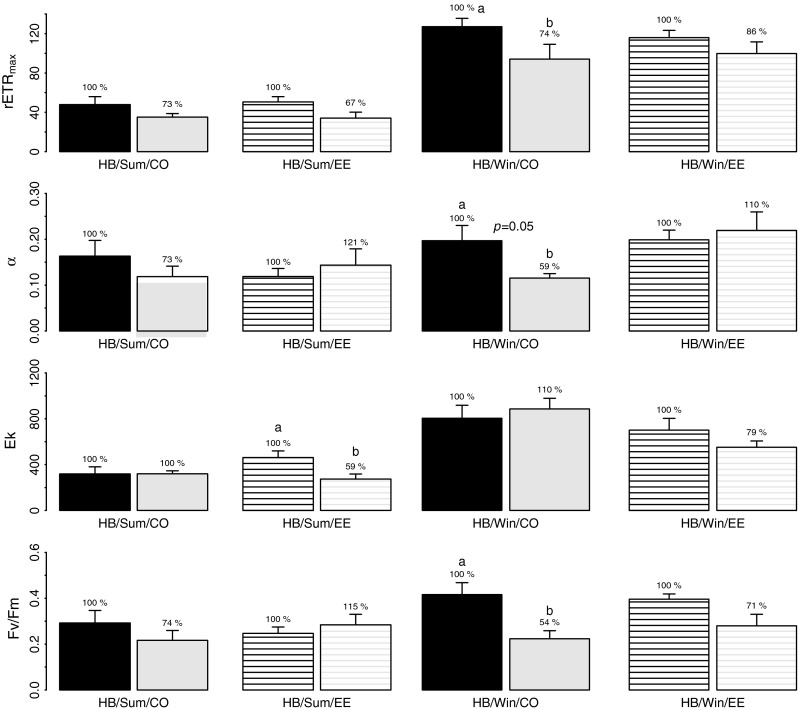
Average *Corallina officinalis* (*CO*—*filled bars*), and *Ellisolandia elongata* (*EE*—*lined bars*) rETR_max_, *α*, *E*_*k*_ and *F*_v_/*F*_m_ at lower shore Heybrook Bay (*HB*) at the start (*black bars*) and end (*light grey bars*) of summer (*Sum*) and winter (*Win*) tidal emersion periods (*n* = 9 ± SE). Percentages demonstrate % change in parameters normalized to start emersion values. *Letters* denote significant differences

*Corallina officinalis*, *C. caespitosa* and *E. elongata* all demonstrated significantly higher rETR_max_ and *E*_*k*_ during winter as compared to summer (Table 4; Figs. 9, 10). *F*_v_/*F*_m_ recorded at the middle and end of tidal emersion at CM was significantly different between seasons for both *C. officinalis* and *C. caespitosa* (post hoc Tukey, *P* < 0.05), while no significant difference in *α* was observed for any species in relation to ‘Season’.

**Table 4:** Analysis of variance of *Corallina* and *Ellisolandia* photophysiology (rETR_max_, *α*, *E*_*k*_ and *F*_v_/*F*_m_) in relation to season, tide and species

	Factor									
	Season		Tide		Species		Season/Tide		Species/Tide	
	*F*_d.f._ and Sig.	Imp. (%)	*F*_d.f._ and Sig.	Imp. (%)	*F*_d.f._ and Sig.	Imp. (%)	*F*_d.f._ and Sig.	Imp. (%)	*F*_d.f._ and Sig.	Imp. (%)
*Combe Martin*										
rETR_max_	***F***_**1**,**104**_ **=** 86.32***	**41**	*F*_2,104_ = 2.82	2.7	*F*_1,104_ = 0.06	0.0	***F***_**2**,**104**_ **=** 6.04**	**5.8**	*F*_2,104_ = 0.35	0.3
*α*	*F*_1,104_ = 1.36	0.9	***F***_**2**,**104**_ **=** 11.75***	**16**	*F*_1,104_ = 0.61	0.4	***F***_**2**,**104**_ **=** 6.80**	**9.7**	*F*_2,104_ = 0.16	0.2
*E*_*k*_	***F***_**1**,**104**_ **=** 84.90***	**41**	***F***_**2**,**104**_ **=** 8.48***	**8.3**	*F*_1,104_ = 1.09	0.5	*F*_2,104_ = 0.84	0.8	*F*_2,104_ = 0.03	0.0
*F*_v_/*F*_m_	***F***_**1**,**104**_ **=** 19.27***	**11**	***F***_**2**,**104**_ **=** 21.14***	**24**	***F***_**1**,**104**_ **=** 4.15*	**2.4**	*F*_2,104_ = 2.67	3.1	*F*_2,104_ = 1.01	1.1
*Heybrook Bay upper*										
rETR_max_	***F***_**1**,**50**_ **=** **43**.**35** ***	**30**	***F***_**2**,**50**_ **=** 21.33***	**30**			*F*_2,50_ = 1.40	1.9		
*α*	*F*_1,50_ = 0.00	0.0	*F*_2,50_ = 1.30	1.6			*F*_2,50_ = 2.12	5.3		
*E*_*k*_	***F***_**1**,**50**_ **=** 73.37***	**47**	***F***_**2**,**50**_ **=** 12.77***	**32**			***F***_**2**,**50**_ **=** 15.38***	**19**		
*F*_v_/*F*_m_	*F*_1,50_ = 0.59	0.5	***F***_**2**,**50**_ **=** 29.56***	**49**			***F***_**2**,**50**_ **=** 4.81*	**8.0**		
*Heybrook Bay lower*										
rETR_max_	***F***_**1**,**72**_ **=** 87.78***	**62**	***F***_**1**,**72**_ **=** 9.63**	**6.9**	*F*_1,72_ = 0.00	0.0	*F*_1,72_ = 0.03	0.0	*F*_1,72_ = 0.04	0.0
*α*	*F*_1,72_ = 2.75	5.4	*F*_1,72_ = 1.30	2.5	*F*_1,72_ = 0.88	1.7	*F*_1,72_ = 0.30	0.6	*F*_1,72_ = 2.96	5.9
*E*_*k*_	***F***_**1**,**72**_ **=** 40.22***	**44**	*F*_1,72_ = 1.68	1.8	*F*_1,72_ = 1.06	1.1	*F*_1,72_ = 0.73	0.8	***F***_**1**,**72**_ **=** 4.64*	**5.1**
*F*_v_/*F*_m_	*F*_1,72_ = 1.60	2.7	***F***_**1**,**7*2***_ **=** 7.90**	**13**	*F*_1,72_ = 0.24	0.4	***F***_**1**,**72**_ **=** 4.51*	**7.6**	*F*_1,72_ = 2.60	4.4

*F* ratios, degrees of freedom and associated significance (*F*_d.f._ and Sig.) (*** *P* < 0.001; ** *P* < 0.01; * *P* < 0.05), and the relative importance of fixed effects (Imp.) are displayed, as determined from linear mixed-effects models. Significant differences are highlighted in bold

Over tidal emersion periods, significant changes in photophysiology parameters were observed at both CM and HB upper/lower shore (Table 4; Figs. 9, 10). At CM, *C. officinalis* and *C. caespitosa* rETR_max_ and *α* demonstrated divergent trends over tidal emersion between seasons, supported by significant interaction between ‘Season’ and ‘Tide’. During summer, both species’ rETR_max_ rose gradually over tidal emersion at CM, though significant increases were restricted to *C. caespitosa*. Concomitantly, *α* increased, showing significantly higher values at the end of emersion, with significantly decreased *E*_*k*_. *C. officinalis* F_v_/*F*_m_ decreased at mid emersion, but recovered by end emersion, with *C. caespitosa* *F*_v_/*F*_m_ also showing significant increase. During winter, *C. officinalis* and *C. caespitosa* photophysiology showed almost identical trends. rETR_max_ decreased from start to mid emersion, showing recovery by end emersion; *α* and *F*_v_/*F*_m_ were significantly decreased at mid emersion in comparison with start and end; and *E*_*k*_ was significantly increased at mid emersion. No significant difference in rETR_max_, *α* or *E*_*k*_ was observed between *C. officinalis* and *C. caespitosa* during summer or winter tidal emersion, while *C. officinalis* *F*_v_/*F*_m_ was significantly increased at the start of tidal emersion during both summer and winter in comparison with *C. caespitosa* (post hoc Tukey, *P* < 0.05).

In upper shore rock pools of HB, *C. officinalis* rETR_max_ demonstrated the opposite trend to that observed during summer at CM, decreasing significantly to 41 % of initial values by the end of tidal emersion. *α* showed no significant change over emersion, while *E*_*k*_ also demonstrated a significant decrease to 55 % of start values. Decreases in *F*_v_/*F*_m_ observed from start to mid emersion showed recovery, though this was not statistically significant. rETR_max_ also decreased over winter tidal emersion, though to a lessor extent than during summer (73 % of start values), accompanied by decreases in *α* and *F*_v_/*F*_m_ and increases in *E*_*k*_. The magnitude of decrease in *F*_v_/*F*_m_ was greater during winter and the direction of change in *E*_*k*_ different between seasons, as highlighted by significant interaction between ‘Season’ and ‘Tide’.

No significant difference in any photophysiology parameter was apparent for *C. officinalis* over summer tidal emersion at lower shore HB, with *E. elongata* only showing decreased *E*_*k*_ from start to end emersion (Table 4; Fig. 10). During winter, significant decrease in *C. officinalis* rETR_max_ was evident from start to end emersion, accompanied by decrease in *α* and *F*_v_/*F*_m_. Conversely, *E. elongata* demonstrated no significant change in any parameter. There was no significant difference between *C. officinalis* and *E. elongata* photophysiology at lower shore HB during either season over tidal emersion, though significant interaction between ‘Species’ and ‘Tide’ was apparent.

**Fig. 11:**
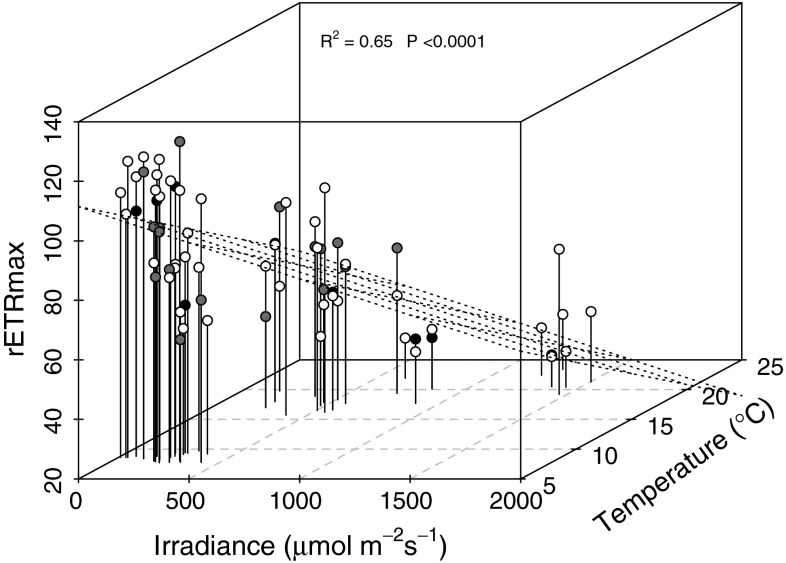
Multiple linear regression of rETR_max_ in relation to irradiance (37 % relative importance) and water temperature (45 % relative importance). *Dashed grid* demonstrates the regression plane. *Corallina officinalis* (*white circles*), *Corallina caespitosa* (*dark grey circles*) and *Ellisolandia elongata* (*black circles*) data are highlighted

*Corallina* and *Ellisolandia* rETR_max_ showed a significant negative linear relationship (*R* ^2^ = 0.65, *P* < 0.001, *n* = 70) with irradiance (as measured) (37 % relative importance) and water temperature (45 % relative importance) (Table 5; Fig. 11). PC1 (16 % relative importance) and PC2 (0 % relative importance) were included in this regression to represent carbonate chemistry, though nonsignificant coefficients were returned for these predictors (Table 5); removal of these predictors did not improve the model quality.

**Table 5:** Multiple linear regression analysis of rETR_max_ in relation to irradiance (expressed as irradiance measured) (Irra.), rock pool water temperature (Temp.), and principal components one (PC1) and two (PC2) from PCA of rock pool water carbonate chemistry

Variable	Intercept	Predictor coefficients ± SE and significance				Relative importance				*R* ^2^	Model *P*	*n*
		Irra.	Temp.	PC1	PC2	Irra.	Temp.	PC1	PC2			
rETR_max_	122.5 ± 7.6	−0.025 ± 0.007**	−2.505 ± 0.693***	−0.509 ± 1.00	0.017 ± 2.50	37 %	45 %	16 %	0 %	0.65	<0.0001	70

Regression coefficients (intercept and predictors) are displayed ± standard error (SE) and with associated significance (*** *P* < 0.001; ** *P* < 0.01; * *P* < 0.05), in addition to the relative importance of predictor variables, associated overall model *R* ^2^ and significance (Model *P*), and the number of observations (*n*)

## Discussion

Recent insights into the identity and distribution of NE Atlantic *Corallina* and *Ellisolandia* allow for updated assessment of species’ ecology underpinned by clear species concepts (Williamson et al. in review, 2009; Brodie et al. 2013). This study represents the first documentation of *C. caespitosa,* as distinguished from *C. officinalis*, photophysiology and contributes to our general understanding of geniculate coralline algal photophysiology in relation to prevailing abiotic conditions. PAM fluorescence and the application of RLC techniques (Ralph and Gademann 2005; Perkins et al. 2006; Burdett et al. 2012) permitted the non-destructive assessment of actual, as opposed to optimal, photosynthetic state of *Corallina* and *Ellisolandia* over summer and winter tidal emersion, facilitating comparison to prevailing irradiance, water temperature and carbonate chemistry conditions. This information is pertinent as research attempts to predict the potential impacts of climate change and OA on calcifying macroalgal species (Harley et al. 2012).

### Abiotic conditions

Our data highlight that *Corallina* and *Ellisolandia* species inhabiting intertidal rock pools are exposed to highly fluctuating irradiance, temperature and carbonate chemistry conditions over both long-term seasonal and short-term tidal emersion periods (Figs. 2, 3, 4, 6). These findings are consistent with previously reported accounts of rock pool habitats (e.g. Ganning 1971; Daniel and Boyden 1975; Truchot and Duhamel-Jouve 1980; Morris and Taylor 1983). Irradiance and temperature increased and were comparatively variable over summer emersion, with irradiance ranging from ca. 487–1,467 μmol photons m^−2^ s^−1^ at HB, with a concomitant increase of ca. 6.4 °C in rock pool temperatures. Lack of significant increase in water temperature over summer or winter tidal emersion at CM was likely due to the larger volumes of rock pools examined at this site (Table 1). Given that smaller and shallower rock pools experience more extreme environmental conditions (Ganning 1971), more stressful conditions may be predicted within upper shore rock pools at HB in comparison with CM. At lower shore HB, rock pool water temperature increases over emersion periods were smaller in magnitude than observed in upper shore pools, consistent with the shorter duration of tidal emersion experienced at lower shore and known gradients of stress experienced across the intertidal (Ganning 1971; Martins et al. 2007).

Significant fluctuations in rock pool carbonate chemistry recorded during the present study were explainable to a high degree (*R* ^2^ = 0.82) by photodose and rock pool water temperature (Figs. 3, 4, 8). These findings are similar to a recent study that demonstrated prediction of DIC fluctuations in a macrophyte meadow using a simple statistical model comprised of three parameters: wind speed, wind direction and PAR (Saderne et al. 2013). Of our two predictors, photodose showed the strongest relative importance in explaining carbonate chemistry dynamics (67 % as compared to 32 % for temperature) which is understandable given the cumulative nature of change in carbonate chemistry over tidal emersion, and the driving role of irradiance for photosynthesis and thus inorganic carbon utilization in rock pools (Truchot and Duhamel-Jouve 1980; Morris and Taylor 1983). Temperature may influence carbonate chemistry both through indirect effects to rock pool inhabitant metabolic rates (Morris and Taylor 1983) and by direct effects to the solubility of CO_2_ in seawater (Wootton et al. 2008).

While *Corallina* and *Ellisolandia* photophysiology did not demonstrate significant relationships with carbonate chemistry across our data, carbonate chemistry dynamics highlighted by this work are important when considering their potential response’s to future OA. As OA proceeds, periodic exposure to high pH conditions may ameliorate some of the negative impacts of OA for calcifying species (Hurd et al. 2011; Anthony et al. 2011; Manzello et al. 2012). In addition, exposure to natural pH variability has been linked to increased resilience of calcifying species to future OA conditions (Wootton et al. 2008; Hofmann et al. 2011; Kelly et al. 2013; Wolfe et al. 2013). Mixed responses of *C. officinalis* and *E. elongata* to future OA conditions have been demonstrated to-date by incubation studies employing static pH conditions (Hofmann et al. 2012, 2013; Noisette et al. 2013; Egilsdottir et al. 2013). To fully elucidate OA impacts to intertidal geniculate coralline species, incubation experiments should be conducted that incorporate natural variability in carbonate chemistry experienced in situ during both daylight and night-time tidal emersion; the latter of which results in opposite trends in carbonate chemistry to those described here (Truchot and Duhamel-Jouve 1980; Egilsdottir et al. 2013).

### Photophysiology

*Corallina* and *Ellisolandia* photophysiology demonstrated patterns of both long-term, seasonal acclimation to changing irradiance and temperature and short-term (hours) acclimation to irradiance changes over tidal emersion during the present study, with the efficiency of short-term acclimation seemingly dependent on the seasonal state. At the seasonal resolution, significantly lower rETR_max_ and *E*_*k*_ were observed for *C. officinalis*, *C. caespitosa* and *E. elongata* during summer, with a negative relationship identified between rETR_max_ and irradiance and temperature across all data (Fig. 11). For most intertidal macroalgae, the quantity of PAR impinging on a plant during summer is often far in excess of that needed to saturate photosynthesis (Franklin and Forster 1997). Excess irradiance can lead to photooxidative damage via increased production of reactive oxygen species, and, in extreme cases, this can cause pigment bleaching and death (Muller et al. 2001). As such, macroalgae must acclimate to changes in light intensity in a manner that optimizes photosynthesis and growth, while controlling for potential stress. Long-term acclimation to changes in light intensity can be achieved via regulation of the size of light-harvesting pigment antennae, through changes in gene expression and proteolysis (Muller et al. 2001).

Reduced rETR_max_ and *E*_*k*_ during summer may therefore reflect seasonal acclimation of *Corallina* and *Ellisolandia* photochemistry as a seasonal response to excess summer irradiance. In this respect, the reverse acclimation to low light conditions must be performed in winter to allow efficient harvesting of reduced irradiance levels. As significantly higher values of rETR_max_ were observed for all species during winter, when minimal irradiance was observed, our data indicate that *Corallina* and *Ellisolandia* are more effective at harvesting and utilizing light energy at low fluence rates, as proposed by Häder et al. (1997) and Häder et al. (2003), who described geniculate coralline species as typical ‘shade plants’.

Over summer tidal emersion at CM, diurnal patterns observed in *Corallina* photophysiology were suggestive of the ability to rapidly acclimate photochemistry to significant changes in irradiance experienced. *C. officinalis* and *C. caespitosa* demonstrated diurnal patterns in *F*_v_/*F*_m_ indicative of photosynthetic downregulation by dynamic photoinhibition, the dissipation of excess light energy as heat (Franklin and Forster 1997). This can serve to prevent long-lasting photooxidative damage caused by excess irradiance, while allowing maintenance of photosynthetic rates (Davison and Pearson 1996; Franklin and Forster 1997; Muller et al. 2001). From start to mid summer emersion, *F*_v_/*F*_m_ decreased or remained reduced when increases in irradiance were observed, followed by complete recovery at the end of emersion when irradiance decreased. Concomitantly, *C. officinalis* and *C. caespitosa* rETR_max_ was maintained and increased, respectively. This confirms that *C. officinalis* and *C. caespitosa* possess the ability to rapidly down-regulate photochemistry in response to excess irradiance over summer tidal emersion, while maintaining electron transport rates. It further demonstrates that down regulation is a dynamic process in these species, easily reversible during summer over the duration of tidal emersion.

Significant decreases in HB upper shore *C. officinalis* rETR_max_ and *E*_*k*_ over summer tidal emersion, however, did not follow the same trend as observed at CM and may indicate electron transport limitation by high pH/low inorganic carbon conditions. While photoinhibition was evident in response to the relatively extreme irradiance prevailing, as evidenced by decreases in *F*_v_/*F*_m_, these decreases were not proportional to rETR_max_ reduction and showed signs of recovery at end emersion, while rETR_max_ did not. Continual decrease in ETR has been observed for *U. intestinalis*, *F. vesiculosus* and *Chondrus crispus* across simulated tidal emersion periods in artificial rock pools, due to parallel increases in pH and decreases in inorganic carbon concentrations (Björk et al. 2004). With the depletion of *p*CO_2_, algae become dependent on HCO_3_^−^ utilization, via conversion to CO_2_ either by extracellular CA (Invers et al. 1997; Badger 2003), or by direct anion exchange-mediated uptake (Larsson and Axelsson 1999). At high pH (8.45–9.3), macroalgal CA activity is often ineffective (Middelboe and Hansen 2007a, b), with consequent decreases in photosynthetic rates (41–78 %) reported for several macroalgal species, compared to rates measured at lower pH (8–8.1) (Israel and Hophy 2002; Middelboe and Hansen 2007a, b; Semesi et al. 2009). While carbonate chemistry changes did not show significant regression to rETR_max_ across all data during the present study, extremes in pH (average pH 9.18 ± 0.08) and, significantly reduced *p*CO_2_ (−96 %) and HCO_3_^−^ (−75 %) concentrations apparent in upper shore HB rock pools at the end of summer emersion, may have contributed to decreases in *C. officinalis* rETR_max_ and warrant further investigation.

Over periods of winter emersion, *Corallina* photophysiology appeared more sensitive to relatively smaller changes in irradiance than those experienced during summer emersion (supporting our proposal of winter acclimation to low irradiance conditions), and down regulation of photochemistry was less effective over tidal emersion periods. At CM, while similar dynamics in *F*_v_/*F*_m_ were observed as during summer, decreases in *F*_v_/*F*_m_ at mid emersion were proportionately larger than those during summer and did not serve to maintain rETR_max_, which was significantly decreased at mid emersion. At HB, both upper and lower shore *C. officinalis* demonstrated significant decreases in *F*_v_/*F*_m_, rETR_max_ and *α* in response to relatively moderate increases in irradiance, with no recovery by the end of emersion in upper shore pools.

Large antennae are necessary for efficient light capture in light limiting conditions, but they can be a liability when light is abundant or excessive (Muller et al. 2001). Low light photoacclimation to winter conditions thus seemed to increase *Corallina* sensitivity to photostress during tidal emersion periods. In addition, photoinhibition was not as effective in maintaining rETR_max_ over winter emersion when irradiance increased. This may be expected given slower acclimation, protein turnover and xanthophyll de-epoxidation under low temperature conditions (Franklin and Forster 1997). However, higher rates of rETR_max_ were still evident overall during winter as compared to summer, suggesting that *Corallina* and *Ellisolandia* achieved a balance between the long-term seasonal and short-term tidal emersion requirements for photoacclimation.

Limited evidence for inter-specific variability in photophysiology was observed during the present study. Though higher *F*_v_/*F*_m_ was observed for *C. officinalis* at the start of tidal emersion in comparison to *C. caespitosa* at CM, patterns in photophysiology were remarkably similar for the two species, with no other differences observed. Similarly, no significant difference in *C. officinalis* and *E. elongata* photophysiology was evident at lower shore HB, though on the whole, *E. elongata* appeared less responsive to changes in abiotic conditions than *C. officinalis*.

Given that the species examined demonstrate both large-scale geographic (Williamson et al. in review; Brodie et al. 2013) and small-scale within-site differences in distribution, differential tolerances to abiotic stressors likely exist. While our data provide information on the photophysiology of *Corallina* and *Ellisolandia* in situ under the influence of highly variable abiotic conditions, laboratory-based analyses of photochemistry using steady-state fluorescence techniques, with control/manipulation of abiotic parameters, are required to disentangle underlying species tolerances. This study provides an initial account of the photophysiology for these keystone species in the context of the environment to which they are adapted in the NE Atlantic.

## References

[CR1] Andersson AJ, Mackenzie FT (2012). Revisiting four scientific debates in ocean acidification research. Biogeosciences.

[CR2] Anthony KRN, Kleypas JA, Gattuso J-P (2011). Coral reefs modify their seawater carbon chemistry—implications for impacts of ocean acidification. Glob Chang Biol.

[CR3] Badger M (2003). The roles of carbonic anhydrases in photosynthetic CO_2_ concentrating mechanisms. Photosynth Res.

[CR4] Bates D, Maechler M, Bolker B, Walker S (2013) lme4: linear mixed-effects models using Eigen and S4. http://CRAN.R-project.org/package=lme4

[CR5] Björk M, Axelsson L, Beer S (2004). Why is *Ulva intestinalis* the only macroalga inhabiting isolated rockpools along the Swedish Atlantic coast?. Mar Ecol Prog Ser.

[CR6] Brodie J, Walker RH, Williamson C, Irvine LM (2013). Epitypification and redescription of *Corallina officinalis* L., the type of the genus, and *C. elongata* Ellis et Solander (Corallinales, Rhodophyta). Cryptogam Algol.

[CR7] Burdett HL, Hennige SJ, Francis FT-Y, Kamenos NA (2012) The photosynthetic characteristics of red coralline algae, determined using pulse amplitude modulation (PAM) fluorometry. Bot Mar. doi:10.1515/bot-2012-0135

[CR8] Cao L, Caldeira K, Jain AK (2007). Effects of carbon dioxide and climate change on ocean acidification and carbonate mineral saturation. Geophys Res Lett.

[CR9] Daniel M, Boyden C (1975). Diurnal variations in physico-chemical conditions within intertidal rockpools. Field Stud.

[CR10] Davison I, Pearson G (1996). Stress tolerance in intertidal seaweeds. J Phycol.

[CR11] Dickson AG, Millero FJ (1987). A comparison of the equilibrium constants for the dissociation of carbonic acid in seawater media. Deep Sea Res A Oceanogr Res Pap.

[CR12] Dickson A, Sabine C, Christian J (2007). Guide to best practices for ocean CO_2_ measurements. PICES Spec Publ.

[CR13] Doney SC (2006). The dangers of ocean acidification. Sci Am.

[CR14] Doney SC (2010). The growing human footprint on coastal and open-ocean biogeochemistry. Science.

[CR15] Egilsdottir H, Noisette F, Noël LM-LJ (2012). Effects of *p*CO_2_ on physiology and skeletal mineralogy in a tidal pool coralline alga *Corallina elongata*. Mar Biol.

[CR16] Eilers PHC, Peeters JCH (1988). A model for the relationship between light intensity and the rate of photosynthesis in phytoplankton. Ecol Model.

[CR17] Franklin LA, Forster RM (1997). The changing irradiance environment: consequences for marine macrophyte physiology, productivity and ecology. Eur J Phycol.

[CR18] Ganning B (1971). Studies on chemical physical and biological conditions in Swedish rockpool ecosystems. Ophelia.

[CR19] Genty B, Briantais JM, Baker NR (1989). The relationship between the quantum yield of photosynthetic electron transport and quenching of chlorophyll fluorescence. Biochim Biophys Acta.

[CR20] Grömping U (2006). Relative importance for linear regression in R: the package relaimpo. J Stat Softw.

[CR21] Häder D-P, Lebert M, Flores-Moya A (1997). Effects of solar radiation on the photosynthetic activity of the red alga *Corallina elongata* Ellis et Soland. J Photochem Photobiol B Biol.

[CR22] Häder D-P, Lebert M, Walter Helbling E (2003). Effects of solar radiation on the patagonian Rhodophyte *Corallina officinalis* (L.). Photosynth Res.

[CR23] Harley CDG, Anderson KM, Demes KW (2012). Effects of climate change on global seaweed communities. J Phycol.

[CR24] Hind KR, Saunders GW (2013). A molecular phylogenetic study of the Tribe Corallineae (Corallinales, Rhodophyta) with an assessment of genus-level taxonomic features and descriptions of novel genera. J Phycol.

[CR25] Hofmann GE, Smith JE, Johnson KS (2011). High-frequency dynamics of ocean pH: a multi-ecosystem comparison. PLoS ONE.

[CR26] Hofmann LC, Yildiz G, Hanelt D, Bischof K (2012). Physiological responses of the calcifying rhodophyte, *Corallina officinalis* (L.), to future CO_2_ levels. Mar Biol.

[CR27] Hofmann LC, Straub S, Bischof K (2013). Elevated CO_2_ levels affect the activity of nitrate reductase and carbonic anhydrase in the calcifying rhodophyte *Corallina officinalis*. J Exp Bot.

[CR28] Hofmann GE, Evans TG, Kelly MW (2014). Exploring local adaptation and the ocean acidification seascape—studies in the California Current Large Marine Ecosystem. Biogeosciences.

[CR29] Hurd CL, Cornwall CE, Currie K (2011). Metabolically induced pH fluctuations by some coastal calcifiers exceed projected 22nd century ocean acidification: a mechanism for differential susceptibility?. Glob Chang Biol.

[CR30] Invers O, Romero J, Perez M (1997). Effects of pH on seagrass photosynthesis: a laboratory and field assessment. Aquat Bot.

[CR31] Irvine LM, Chamberlain YM (1994) Seaweeds of the British Isles, vol 1. Rhodophyta. Part 2B. Corallinales, Hildenbrandiales. HMSO, London

[CR32] Israel A, Hophy M (2002). Growth, photosynthetic properties and Rubisco activities and amounts of marine macroalgae grown under current and elevated seawater CO_2_ concentrations. Glob Chang Biol.

[CR33] Johansen HW (1981) Coralline algae: a first synthesis. CRC Press, Boca Raton, Florida, p 239. ISBN 0-8493-5261-4

[CR34] Jones CG, Lawton JH, Shachak M (1994). Organisms as ecosystem engineers. Oikos.

[CR35] Kelly MW, Padilla-Gamiño JL, Hofmann GE (2013). Natural variation and the capacity to adapt to ocean acidification in the keystone sea urchin *Strongylocentrotus purpuratus*. Glob Chang Biol.

[CR36] Larsson C, Axelsson L (1999). Bicarbonate uptake and utilization in marine macroalgae. Eur J Phycol.

[CR37] Lobban CS, Harrison PJ (1994). Seaweed ecology and physiology.

[CR38] Luning K (1990) Seaweeds: their environment, biogeography and ecophysiology. Wiley, New York, p 527

[CR39] Manzello DP, Enochs IC, Melo N (2012). Ocean acidification refugia of the Florida Reef Tract. PLoS ONE.

[CR40] Martins GM, Hawkins SJ, Thompson RC, Jenkins SR (2007). Community structure and functioning in intertidal rock pools: effects of pool size and shore height at different successional stages. Mar Ecol Prog Ser.

[CR41] Mehrbach C, Culberson CH, Hawley JE, Pytkowicz RM (1973). Measurement of the apparent dissociation constants of carbonic acid in seawater at atmospheric pressure. Limnol Oceanogr.

[CR42] Middelboe A, Hansen P (2007). High pH in shallow-water macroalgal habitats. Mar Ecol Prog Ser.

[CR43] Middelboe A, Hansen P (2007). Direct effects of pH and inorganic carbon on macroalgal photosynthesis and growth. Mar Biol Res.

[CR44] Morris S, Taylor AC (1983). Diurnal and seasonal-variation in physicochemical conditions within intertidal rock pools. Estuar Coast Shelf Sci.

[CR45] Muller P, Xiao-ping L, Niyogi KK (2001). Update on photosynthesis non-photochemical quenching. A response to excess light energy 1. Plant Physiol.

[CR46] Nelson WA (2009). Calcified macroalgae—critical to coastal ecosystems and vulnerable to change: a review. Mar Freshw Res.

[CR47] Noisette F, Egilsdottir H, Davoult D, Martin S (2013). Physiological responses of three temperate coralline algae from contrasting habitats to near-future ocean acidification. J Exp Mar Bio Ecol.

[CR48] Perkins RG, Mouget J-L, Lefebvre S, Lavaud J (2006). Light response curve methodology and possible implications in the application of chlorophyll fluorescence to benthic diatoms. Mar Biol.

[CR49] Perkins RG, Kromkamp JC, Serôdio J et al. (2010) Chlorophyll a fluorescence in aquatic sciences: methods and applications. doi:10.1007/978-90-481-9268-7

[CR50] Pierrot D, Lewis E, Wallace DWR (2006) MS Excel program developed for CO_2_ system calculations. ORNL/CDIAC-105a. Carbon Dioxide Information Analysis Center, Oak Ridge National Laboratory, US Department of Energy, Oak Ridge. doi:10.3334/CDIAC/otg.CO2SYS_XLS_CDIAC105a

[CR54] R Core Team (2013) R: a language and environment for statistical computing

[CR51] Ralph PJ, Gademann R (2005). Rapid light curves: a powerful tool to assess photosynthetic activity. Aquat Bot.

[CR52] Saderne V, Fietzek P, Herman PMJ (2013). Extreme variations of *p*CO_2_ and pH in a macrophyte meadow of the Baltic Sea in summer: evidence of the effect of photosynthesis and local upwelling. PLoS ONE.

[CR53] Semesi IS, Kangwe J, Björk M (2009). Alterations in seawater pH and CO_2_ affect calcification and photosynthesis in the tropical coralline alga, Hydrolithon sp. (Rhodophyta). Estuar Coast Shelf Sci.

[CR55] Tremblay A, Ransijn J (2013) LMERConvenienceFunctions: A suite of functions to back-fit fixed effects and forward-fit random effects, as well as other miscellaneous functions. http://cran.r-project.org/web/packages/LMERConvenienceFunctions/index.html

[CR56] Truchot J-P, Duhamel-Jouve A (1980). Oxygen and carbon dioxide in the marine intertidal environment: diurnal and tidal changes in rockpools. Respir Physiol.

[CR57] Venables WN, Ripley BD (2002). Modern applied statistics with S.

[CR58] Walker RH, Brodie J, Russell S (2009). Biodiversity of coralline algae in the northeastern Atlantic including *Corallina caespitosa*. nov. (Corallinoideae, Rhodophyta). J Phycol.

[CR61] Williamson C, Walker R, Robba L et al (in review) Towards resolution of diversity in *Corallina* (Corallinales, Rhodophyta) and related genera. Phycologia

[CR59] Wolfe K, Dworjanyn SA, Byrne M (2013). Effects of ocean warming and acidification on survival, growth and skeletal development in the early benthic juvenile sea urchin (Heliocidaris erythrogramma). Glob Chang Biol.

[CR60] Wootton JT, Pfister CA, Forester JD (2008). Dynamic patterns and ecological impacts of declining ocean pH in a high-resolution multi-year dataset. Proc Natl Acad Sci USA.

